# Supplementary material for: Lasing in dark and bright modes of a finite-sized plasmonic lattice
Source: Nat Commun. 2017 Jan 3;8:13687. doi: 10.1038/ncomms13687 (PMC5216126; doi:10.1038/ncomms13687)
Supplement: Supplementary Information — Supplementary Figures 1-9, Supplementary Notes 1-4 and Supplementary References. [file ncomms13687-s1.pdf]

Supplementary information
Lasing in dark and bright modes of a finite-sized plasmonic lattice

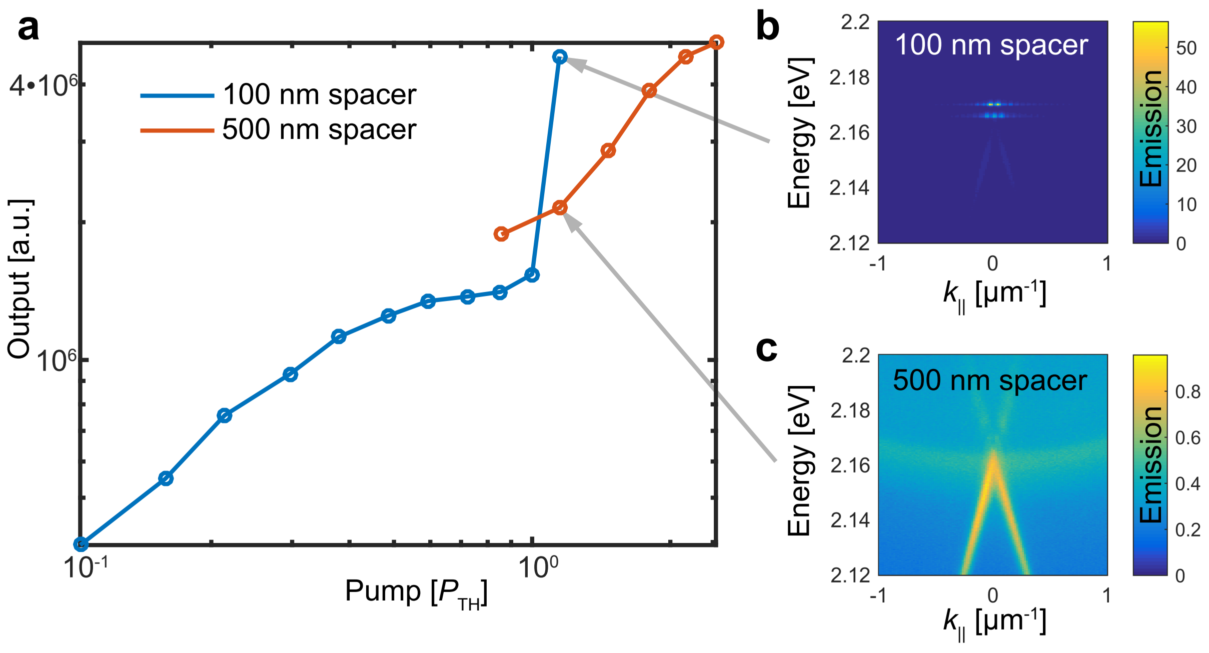

Supplementary Figure 1. *Control experiment results where a dielectric spacer layer was added on top of the nanoparticle array.* Luminescence intensity as a function of the pump power (a) for samples having a PVA spacer layer of 100 nm (blue) and 500 nm (red). The pump powers are normalized with the threshold of the sample having the 100 nm PVA layer. Luminescence as a function of in-plane wave vector and energy at P_{TH} for a PVA spacer of 100 nm (b) and 500 nm (c). The colorscale represents the observed emission intensity in (b) and (c) at the slightly above the threshold pump power P_{TH}.

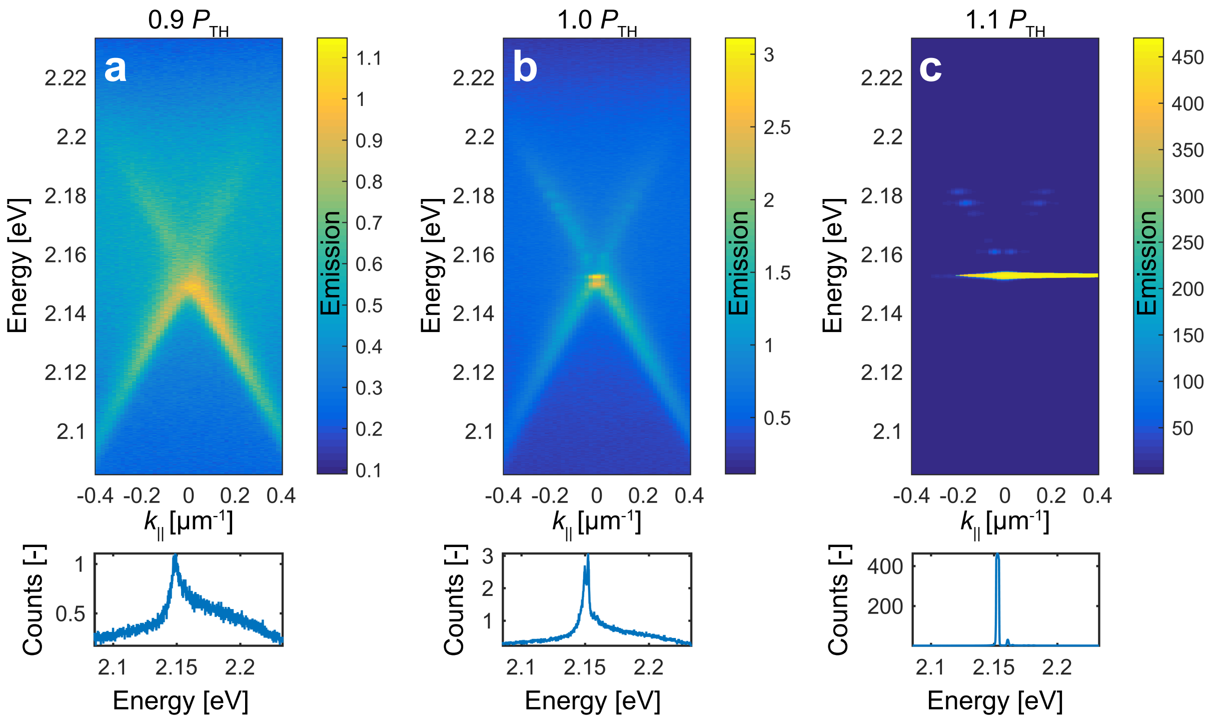

Supplementary Figure 2. *Control experiment results where a fraction of the nanoparticles are removed.* (a-c) Luminescence data for a sample with 0 % of particles removed and with pump powers 0.9 P_{TH}, 1.0 P_{TH} and 1.1 P_{TH}, respectively. The colorscale represents the observed emission intensity. In (c), the spectrometer CCD was strongly saturated by the laser emission, causing the apparent spreading along the horizontal axis. Below each plot are shown the crosscuts in energy at $k_{||} = 0$.

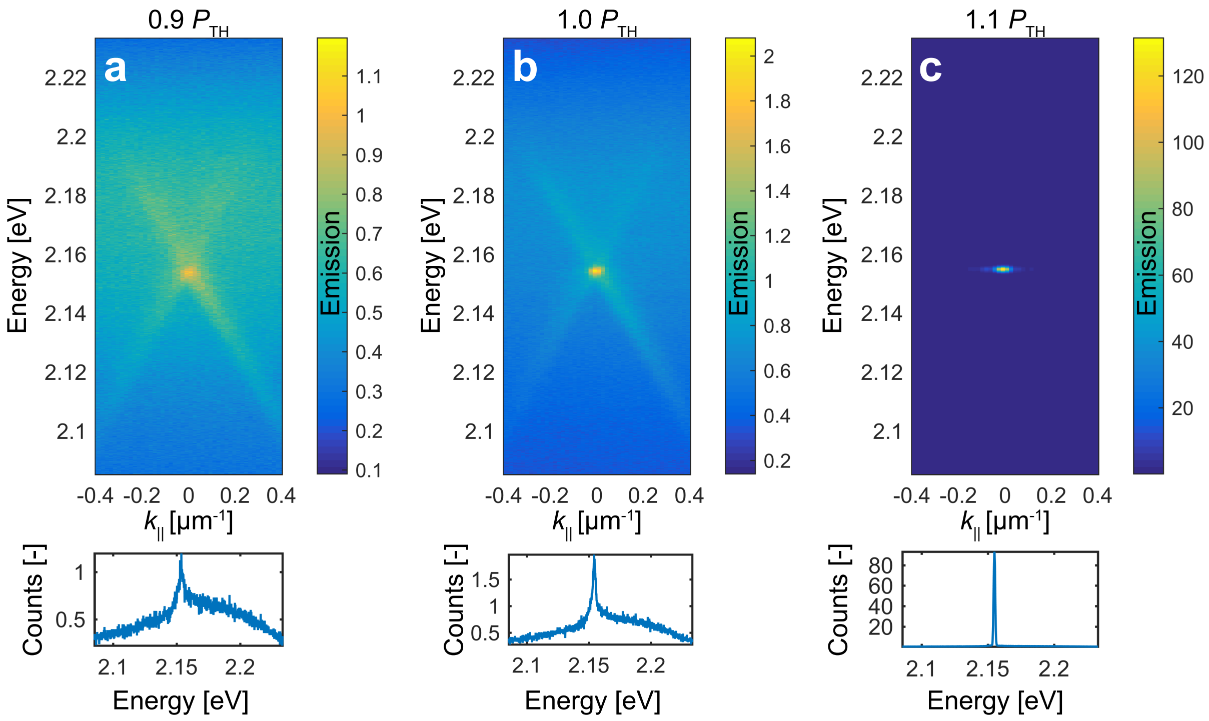

Supplementary Figure 3. *Control experiment results where a fraction of the nanoparticles are removed.* Luminescence data for a sample with 50 % of particles removed from random positions on the array. The pump powers were (a) 0.9 P_{TH}, (b) 1.0 P_{TH} and (c) 1.1 P_{TH}. The colorscale represents the observed emission intensity. Below each plot are shown the crosscuts in energy at $k_{||} = 0$.

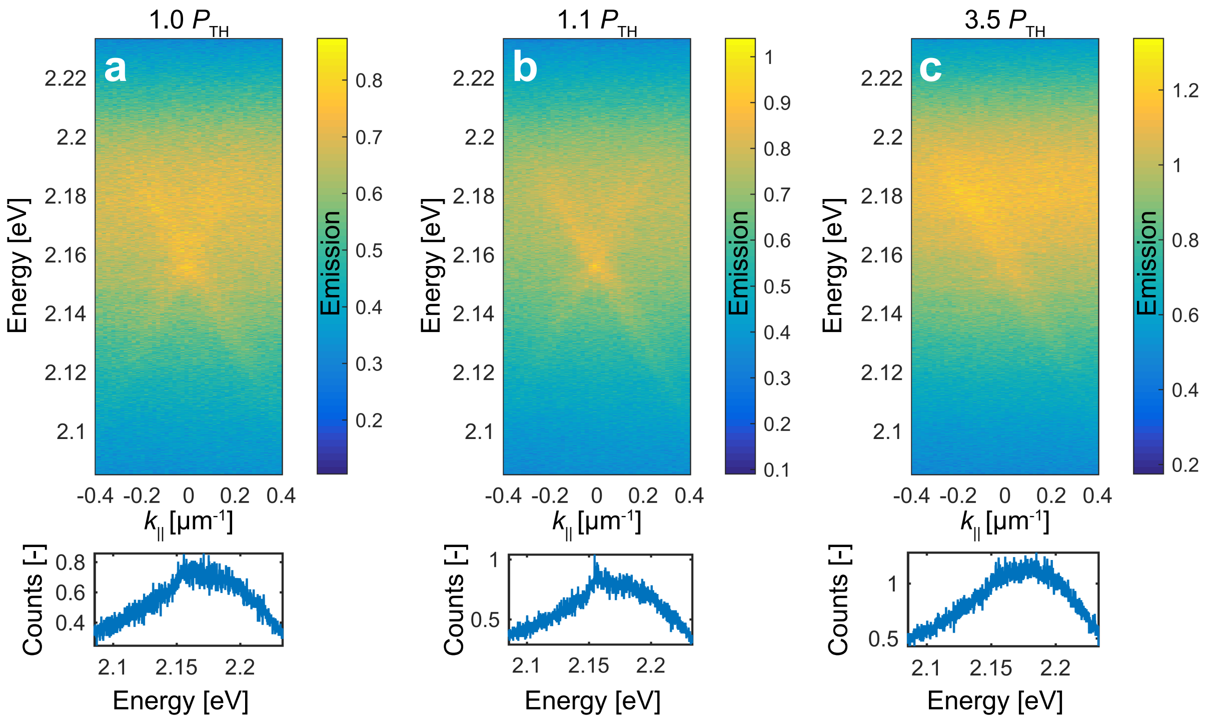

Supplementary Figure 4. *Control experiment results where a fraction of the nanoparticles are removed.* Luminescence data for a sample with 75 % of particles removed from random positions on the array. The pump powers were (a) 1.0 P_{TH}, (b) 1.1 P_{TH} and (c) 3.5 P_{TH}. The colorscale represents the observed emission intensity. Below each plot are shown the crosscuts in energy at $k_{||} = 0$. No lasing was observed.

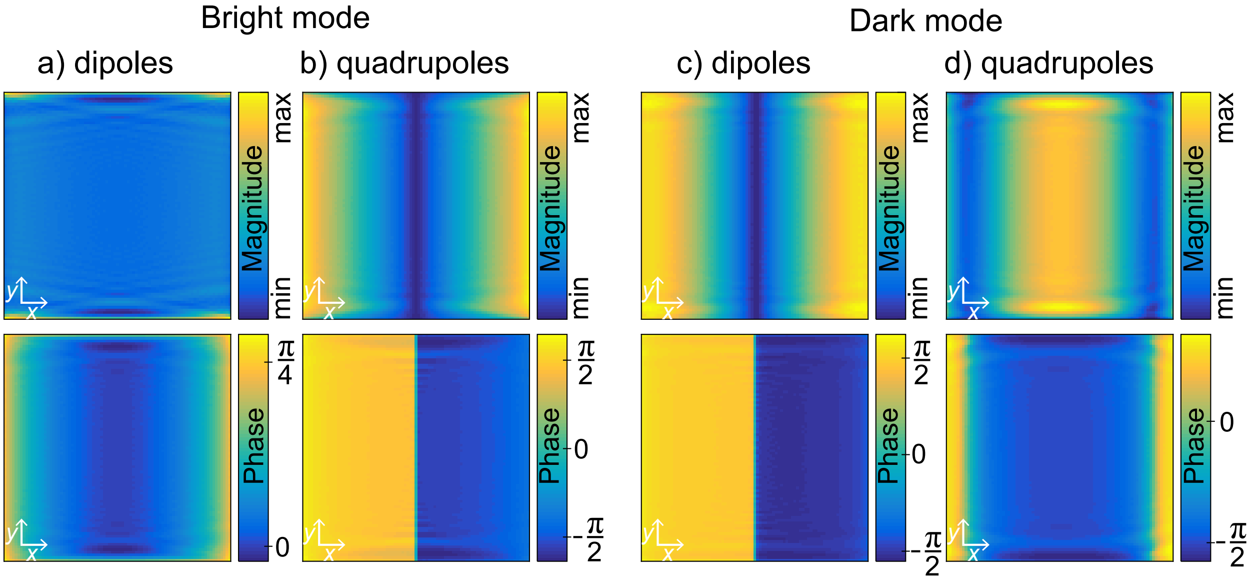

Supplementary Figure 5. *Electric dipole and quadrupole response of a* 85×85 *nanoparticle lattice calculated with the multiple scattering T-matrix method.* The nanoparticles are excited with a y-polarized plane wave coming from the normal direction (a, b), or a combination of two y-polarized plane wave coming from the opposite angles so that the exciting field has a π-phase difference between the vertical edges (c, d). The upper row shows the polarization magnitude, the lower row shows its phase. The spacing between particles is 375 nm and the frequencies correspond to the bright (a, b) and dark (c, d) modes.

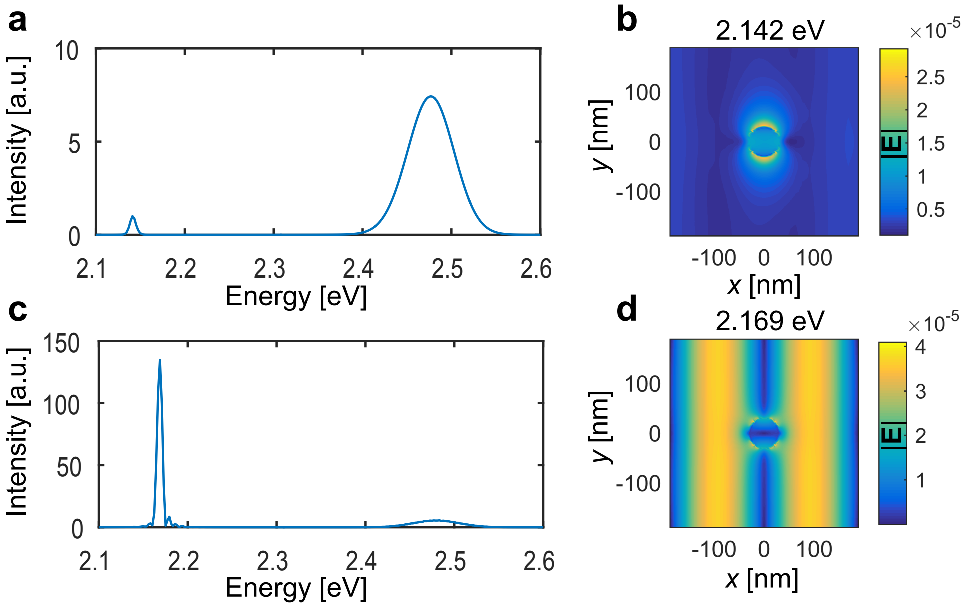

Supplementary Figure 6. *Normalized lasing spectra and corresponding field profiles obtained from FDTD simulations with gain.* The results for the bright SLR mode are shown in (a-b) and for the dark SLR mode in (c-d). The colorscales represent the electric field profiles at the lasing frequency in a single unit cell. The pump power was set to $0.63\,\mathrm{mJ} \cdot \mathrm{cm}^{-2}$ in the simulation. For this pump power, both modes are well above the lasing threshold, see Fig. 2 g of the main text, but the dark mode output power is about an order of magnitude higher than that of the bright mode. In (a), the spectrum is calculated from the electric fields at the bottom left corner of the unit cell. At this location, the dark mode field profile (d) has a node, and thus it is not visible in the calculated spectra. The smaller peak at $2.142\,\mathrm{eV}$ corresponds to the bright SLR mode, for which the field profile is shown in (b). The larger peak at $2.48\,\mathrm{eV}$ comes from the pump pulse. The dark SLR mode lasing spectra is shown in (c), and the electric field profile at the lasing peak energy $2.169\,\mathrm{eV}$ is shown in (d). The spectra is calculated 1/4th of the unit cell away from the corner along the horizontal axis. At this location, the bright mode has a node in its field profile, and thus is not visible in the calculated spectra. The bright SLR mode peak has been normalized to be 1 in the intensity scales.

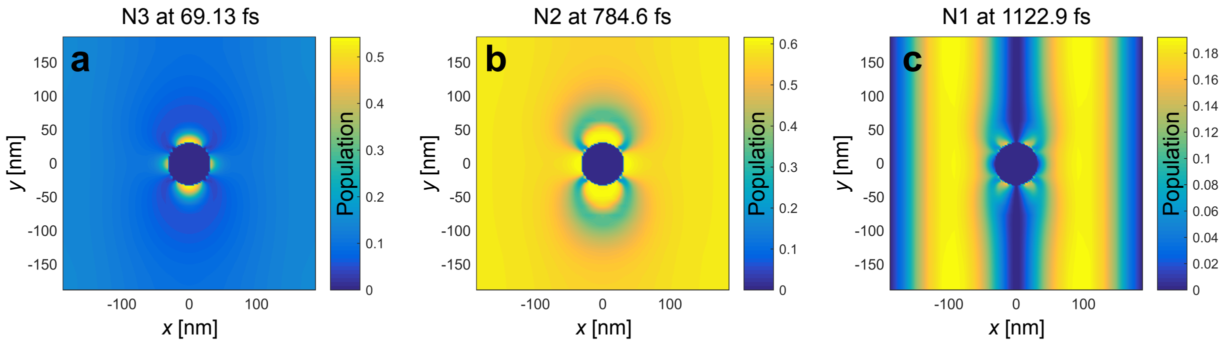

Supplementary Figure 7. *Snapshots of the populations at different points of the simulation in different levels of the 4-level gain medium.* The colorscale repesents the fraction of molecules in a specific state at a specific time of the simulation run. In (a) the highest energy state population is shown. The pump pulse is starting to interact with the structure, showing how the pump interacts with the gain medium both directly, and through the surface plasmon resonance of the nanoparticles. In (b), the upper lasing state population is shown, after the population pumped to the highest energy state has decayed into the upper lasing state, but before lasing in the SLR mode starts. In (c) is the lower lasing state population after the onset of lasing in the SLR mode. The lower lasing state population shows where stimulated emission is taking place, and this matches well with the field profile of the dark SLR mode shown in Supplementary Fig. **6** (d). The pump power is chosen to be below the threshold for the bright SLR mode lasing (field profile shown in Supplementary Fig. **6** (b)).

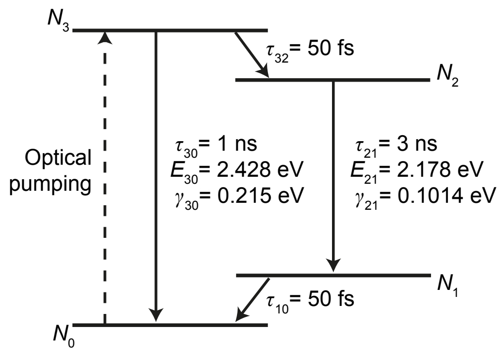

Supplementary Figure 8. *Energy level diagram and the parameters used for modelling the dye molecules in FDTD simulations.* The transition energies E_{30} and E_{21}, and the full-widths at half-maximum of these transitions γ_{30} and γ_{21} are obtained from absorption and emission measurements of the Rhodamine 6G solution used in the experiments. In the beginning of a simulation, $N_0 = 1$. Then a fraction of the molecules gets excited by a pump pulse, causing level 3 to gain population. As the transition from level 3 to level 2 is much faster than the transition form level 3 to 0, the excitations quickly decay to level 2. This causes a population inversion for the transition from level 2 to 1, which supplies the necessary gain for lasing in the SLR modes.

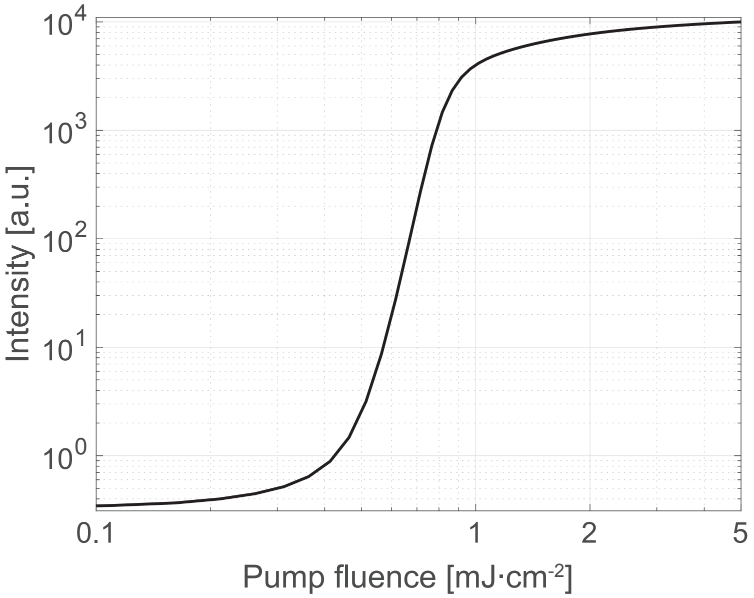

Supplementary Figure 9. *The threshold behaviour as predicted by the neo-classical lasing model.*

Supplementary Note 1. Control experiments for the observed lasing action

The crucial role of the plasmonic near fields for the lasing action is also demonstrated by our control experiment where the gain medium is spatially separated from the array by a poly vinyl alcohol (PVA) layer: A sample with a 100 nm PVA spacer layer still exhibited lasing action with a clear threshold, but a sample with a 500 nm PVA layer did not, see Supplementary Fig. **1**. Supplementary Fig. **1** (b) displays clear lasing in the 100 nm spacer layer sample, while the 500 nm spacer layer sample in Supplementary Fig. **1** (c) shows emission in a broad range of energy and momentum values. After removing the PVA layer and consequent replacement of the gain medium, however, the sample recovered its lasing action.

The lasing action in our samples is different from distributed feedback lasers consisting of a waveguide, gain medium and a periodic metallic structure. In such systems, the periodic structure merely provides the feedback for the waveguide mode. In our case, however, the refractive index of the gain medium is closely matched with the substrate and the cover slip, so the structure is not expected to support any waveguide modes, but rather only the surface lattice resonance (SLR). In [1], waveguide modes were hybridized with SLR modes, and up to 95 % of the particles could be removed while still preserving the lasing action. In contrast, our samples exhibit no lasing if 75 % (or more) of the particles are removed. In Supplementary Figs. **2**–**4** are shown luminescence data for samples having 0 %, 50 % and 75 % of particles removed, respectively. For each sample, we plot the luminescence data with pump power below, at and above the threshold value P_{TH} which is the threshold for the sample with 0 % of particles removed. A sample having 50 % of particles removed still lases (Supplementary Fig. **3**). A sample having 75 % of particles removed did not lase at all, even with pump powers up to $3.5\,P_{\mathrm{TH}}$ (Supplementary Fig. **4**).

A sample with the same number, shape and area of nanoparticles but in random positions shows no lasing either, even when pumped with powers up to $P = 3.5\,P_{\mathrm{TH}}$, where P_{TH} is the threshold for the sample with no particles removed. This rules out random lasing as the possible origin for the observed lasing action.

Supplementary Note 2. Coupled dipole and multipole scattering simulations

We simulated the linear response of our nanoparticle arrays with the multiple-scattering T-matrix method, principles of which we briefly sketch in the following. The technical details can be found in the literature [2–5].

Scattering of a time-harmonic electromagnetic incident field on a single scatterer in otherwise isotropic background medium can be described in terms of the regular $\mathbf{M}_{mn}^{(1)}, \mathbf{N}_{mn}^{(1)}$ and outgoing $\mathbf{M}_{mn}^{(3)}, \mathbf{N}_{mn}^{(3)}$ vector spherical wave functions (VSWFs), which are the solutions of vector Helmholtz equation. The outgoing VSWFs $\mathbf{M}_{mn}^{(3)}$ and $\mathbf{N}_{mn}^{(3)}$ correspond to the field emitted by harmonically oscillating magnetic and electric multipoles, respectively. Outside a sphere surrounding the scatterer, the electromagnetic field is the sum of the incident and

scattered field

$$\mathbf{E} = \mathbf{E}_{\text{inc}} + \mathbf{E}_{\text{scat}} \tag{1}$$

$$\mathbf{E}_{\text{inc}}(\mathbf{r}) = \sum_{n=1}^{\infty} \sum_{m=-n}^{n} \left[q_{mn} \mathbf{M}_{mn}^{(1)}(kr, \theta, \phi) + p_{mn} \mathbf{N}_{mn}^{(1)}(kr, \theta, \phi) \right], \tag{2}$$

$$\mathbf{E}_{\text{scat}}(\mathbf{r}) = \sum_{n=1}^{\infty} \sum_{m=-n}^{n} \left[b_{mn} \mathbf{M}_{mn}^{(3)}(kr, \theta, \phi) + a_{mn} \mathbf{N}_{mn}^{(3)}(kr, \theta, \phi) \right], \tag{3}$$

where k is the wavenumber of the field in the background medium at a given frequency. Because of the linearity of Maxwell's equations, there is a linear relation between the expansion coefficients of the incident and scattered parts

$$a_{mn} = \sum_{n'=1}^{\infty} \sum_{m'=-n'}^{n'} \left[T_{mnm'n'}^{EE} p_{m'n'} + T_{mnm'n'}^{EM} q_{m'n'} \right], \tag{4}$$

$$b_{mn} = \sum_{n'=1}^{\infty} \sum_{m'=-n'}^{n'} \left[T_{mnm'n'}^{ME} p_{m'n'} + T_{mnm'n'}^{MM} q_{m'n'} \right]. \tag{5}$$

The coefficients $T_{mnm'n'}^{tt'}$ are the elements of the T-matrix which completely characterizes the linear electromagnetic response of a single scatterer.

Using the translation addition theorem, the outgoing VSWFs can be re-expanded into the regular VSWFs in a spherical coordinate system with a different origin (labeled with j, as opposed to the original system which we label with l)

$$\mathbf{M}_{\mu\nu}^{(3)}(\mathbf{r}_l) = \sum_{nm} \left[A_{mn\mu\nu}^{(1)}(\mathbf{r}_{lj}) \mathbf{M}_{mn}^{(1)}(\mathbf{r}_j) + B_{mn\mu\nu}(\mathbf{r}_{lj}) \mathbf{N}_{mn}^{(1)}(\mathbf{r}_j) \right], \tag{6}$$

$$\mathbf{N}_{\mu\nu}^{(3)}(\mathbf{r}_l) = \sum_{mn} \left[B_{mn\mu\nu}^{(1)}(\mathbf{r}_{lj}) \mathbf{M}_{mn}^{(1)}(\mathbf{r}_j) + A_{mn\mu\nu}(\mathbf{r}_{lj}) \mathbf{N}_{mn}^{(1)}(\mathbf{r}_j) \right], \tag{7}$$

$$|\mathbf{r}_j| < |\mathbf{r}_{lj}|, \tag{8}$$

where the vector $\mathbf{r}_{lj} = \mathbf{r}_l - \mathbf{r}_j$ connects the origins of both systems. If a second scatterer is present in the centre of the new coordinate system, the field scattered from the first scatterer incident onto the second one can be obtained. This way one can proceed with an arbitrary number of scatterers, leading to a linear system that, in matrix notation, is given by

$$\begin{bmatrix} \mathbf{a}^j \\ \mathbf{b}^j \end{bmatrix} = \mathbf{T}^j \left(\begin{bmatrix} \mathbf{p}^{j0} \\ \mathbf{q}^{j0} \end{bmatrix} + \sum_{l \neq j} \begin{bmatrix} \mathbf{A}(k\mathbf{r}_{lj}) & \mathbf{B}(k\mathbf{r}_{lj}) \\ \mathbf{B}(k\mathbf{r}_{lj}) & \mathbf{A}(k\mathbf{r}_{lj}) \end{bmatrix} \begin{bmatrix} \mathbf{a}^l \\ \mathbf{b}^l \end{bmatrix} \right), \tag{9}$$

which can be readily solved to obtain the solution for the scattering problem on a nanoparticle array.

Expressions for the translation coefficients $A_{mn\mu\nu}(\mathbf{r}_{lj}), B_{mn\mu\nu}(\mathbf{r}_{lj})$ as well as the expansion coefficients p_{mn}, q_{mn} for the initial exciting plane wave can be found in the literature [3, 4]. The only missing part is then finding the T-matrix for a single nanoparticle, which we compute by the boundary element method [6, 7].

The simulations presented in the article were performed up to the quadrupole order, i.e. only the elements with $n = 1, 2$ were included in the calculation. Including higher-order

terms while keeping the array size reasonably large is problematic mainly due to the memory limitations.

In Supplementary Fig. **5** we show the simulated electromagnetic response of a 85×85 particle lattice at both bright and dark mode energies. We note that experimentally we observed the bright mode lasing perpendicularly to the array plane, while the dark mode had two beams coming out with slight deviation from the sample normal (see also Fig. 2 c of the manuscript). Therefore, in order to excite these modes with an external plane wave, one has to use direct incidence for the bright mode, and two plane waves with a slightly tilted angle and a π phase shift for the dark mode.

For the bright mode, the lattice is excited by a y-polarized plane wave coming from the normal direction: the electric dipole moments obtain approximately constant magnitude and phase across the array (Supplementary Fig. **5** (a)). At the center of the array, the standing wave due to two counter propagating radiation fields creates a standing wave pattern which has an antinode at each particle location. Due to symmetry, such a standing wave cannot excite the quadrupole moment into the center particles, resulting to a minimum magnitude of the quadrupolar moment at the array center (Supplementary Fig. **5** (b)). At the edges, however, only one of the counter propagating is present. Such a wave can excite the quadrupole moment to finite size of the particles (diameter 60 nm). The left and right propagating plane waves are out of phase by π, creating an abrupt phase shift at the center of the array (Supplementary Fig. **5** (b) bottom).

We then inspect the response at the dark mode energy (Supplementary Figs. **5** (c, d)), where we use an incidence angle of $\arcsin(\pi/kd_x) = 5.87 \times 10^{-3}$ rad. Here d_x is the array size. At the center of the array, the two counter propagating plane waves are of equal magnitude and out of phase by π, creating a standing wave node at each particle location. Due to symmetry, such a wave cannot excite the dipolar moment into the particles, resulting to 1) minimum of the dipole moment and 2) maximum of the quadrupole moment at the center of the array. At the edges, only one of the counter propagating plane waves is present, therefore exciting the dipole moment of the particle. As the counter propagating plane waves are out of phase by π, the dipole moment distribution undergoes an abrupt phase shift at the center of the array.

One can summarize the results in the following way: 1) For the bright mode, the dipolar moment maximizes at the center and has roughly a constant phase across the array. 2) Quadrupolar moments minimize, and undergo a π phase shift at the center of the array. 3) For the dark mode, the opposite happens, i.e., dipoles minimize and undergo a π phase shift at the center of the array and 4) quadrupoles maximize at the center and have roughly constant phase across the array.

Supplementary Note 3. FDTD simulations with an active gain medium

The response of the dye molecules placed on top of the samples in the experiments was simulated using the FDTD method, similar to Refs. [8] and [9]. We use Lumerical's FDTD Solutions software, version 8.16.903 for the simulations. The dye molecules are modelled as 4-level systems, which are coupled in time domain to the electric fields of the nanoparticle arrays. The dye is placed as a 150nm thick film on top of the particles. The 4-level energy diagram, and parameters used for the simulations are shown in Supplementary Fig. **8**. The dye transition energies and full-width half-maximum values are obtained from absorption and emission measurements of the 31 mM Rhodamine 6G dye solution used in the experiments.

The density of the emitters is $1.867 \cdot 10^{19}\,\mathrm{cm}^{-3}$, corresponding to the 31 mM concentration used in the experiments.

In the beginning of the simulation, all of the dye molecules are in the ground state, $N_0 = 1$. We then use a 30 fs pump pulse, centered at 500 nm (2.48 eV), to excite the molecules. The pump is linearly polarized along the y-axis. Initially this pulse excites a fraction of the molecules to state N_3, which then relax to state N_2, creating a population inversion. After a sufficient population inversion is reached, stimulated emission to the dark or bright SLR modes quickly brings down the population of the state N_2. Emission spectrum obtained for the dark and bright modes from the FDTD simulations are shown in Supplementary Fig. **6**, together with the field profiles at peak emission energies from the same simulation run. The energies of these modes are slightly shifted compared to the simulations without the active media (see Fig. 1 of the main text) through a change in the refractive index, which depends on the input power. The lattice period is 375 nm for both x and y directions.

Snapshots of the level populations observed with a pump power of $0.31\,\mathrm{mJ} \cdot \mathrm{cm}^{-2}$ at different times are shown in Supplementary Fig. **7**. The level populations are monitored in the x-y plane of the simulation, at the middle height of the nanoparticles. In Supplementary Fig. **7** (a) is shown the population of state N_3 slightly before the pump pulse reaches its maximum intensity. Due to the localized surface plasmon resonance in the nanoparticles, the dye molecules close to the particle are pumped the hardest. In Supplementary Fig. **7** (b) the upper lasing level N_2 population is shown after the pump has passed through the structure, and the population in level N_3 has decayed into level N_2. The structure does not lase yet. Slightly away from the nanoparticle, a band with lower amount of excited molecules is observed. This is due to overpumping the dye molecules close to the particle through the surface plasmon resonance. In Supplementary Fig. **7** (c) is shown the lower lasing state population N_1 after the structure has started to lase in the dark SLR mode. The lower lasing state population shows where stimulated emission has occurred, and this can be compared to the field profile of the dark SLR mode shown in Supplementary Fig. **6** (d).

Supplementary Note 4. Neo-classical model of lasing

We model lasing in our system also along the neo-classical lines as explained in Ref. [10]. All nano-particles are assumed to be equal and well described by the same dipole. The array structure influences the effective polarizability via the coupled dipole approximation (CDA). Oscillating dipoles can then give rise to the lasing field when they are coupled with a gain medium. We model the Rhodamine 6G gain medium as a four-level system with parameters taken from Ref. [11]. The transition between the ground state and highest state is driven by a coherent laser pulse similar to experiments. We assume 10 % active molecules, a Purcell factor of 2, and SLR loss rate of $\gamma_{SLR} = 3\,\mathrm{meV}$. In Supplementary Fig. **9** we demonstrate the resulting threshold behaviour. These results show that a neo-classical model that describes the SLR modes in a simplified manner is sufficient to produce the overall threshold behaviour. However, it is not able to describe lasing in the dark and bright modes included in the full FDTD-based model in Supplementary Note 3.

Supplementary References.

[1] Schokker, A. H. & Koenderink, A. F. Statistics of Randomized Plasmonic Lattice Lasers. *ACS Photonics* **2**, 1289–1297 (2015).

[2] Mishchenko, M. I., Travis, L. D. & Macke, A. T-matrix method and its applications. In *Light Scattering by Nonspherical Particles: Theory, Measurements, and Applications*, 147–172 (Academic Press, 1999).

[3] Xu, Y.-l. Calculation of the Addition Coefficients in Electromagnetic Multisphere-Scattering Theory. *J. Comput. Phys.* **127**, 285–298 (1996).

[4] Xu, Y.-l. Radiative scattering properties of an ensemble of variously shaped small particles. *Physical Review E* **67**, 046620 (2003).

[5] Taylor, J. M. *Optical Binding Phenomena: Observations and Mechanisms* (Springer Science & Business Media, 2011).

[6] Homer Reid, M. T. & Johnson, S. G. Efficient Computation of Power, Force, and Torque in BEM Scattering Calculations. *IEEE Trans. Antennas Propagat.* **63**, 3588–3598 (2015).

[7] http://github.com/homerreid/scuff-EM.

[8] Dridi, M. & Schatz, G. C. Model for describing plasmon-enhanced lasers that combines rate equations with finite-difference time-domain. *J. Opt. Soc. Am. B* **30**, 2791 (2013).

[9] Dridi, M. & Schatz, G. C. Lasing action in periodic arrays of nanoparticles. *J. Opt. Soc. Am. B* **32**, 818 (2015).

[10] Martikainen, J.-P., Hakala, T. K., Rekola, H. T. & Törmä, P. Modelling lasing in plasmonic nanoparticle arrays. *J. Opt.* **18**, 024006 (2016).

[11] Cuerda, J., Rüting, F., García-Vidal, F. J. & Bravo-Abad, J. Theory of lasing action in plasmonic crystals. *Phys. Rev. B* **91**, 041118 (2015).
